# Early Expression of Hypocretin/Orexin in the Chick Embryo Brain

**DOI:** 10.1371/journal.pone.0106977

**Published:** 2014-09-04

**Authors:** Kyle E. Godden, Jeremy P. Landry, Natalya Slepneva, Paola V. Migues, Maria Pompeiano

**Affiliations:** Department of Psychology, McGill University, Montreal, Quebec, Canada; University of Rouen, France, France

## Abstract

Hypocretin/Orexin (H/O) neuropeptides are released by a discrete group of neurons in the vertebrate hypothalamus which play a pivotal role in the maintenance of waking behavior and brain state control. Previous studies have indicated that the H/O neuronal development differs between mammals and fish; H/O peptide-expressing cells are detectable during the earliest stages of brain morphogenesis in fish, but only towards the end of brain morphogenesis (by ∼85% of embryonic development) in rats. The developmental emergence of H/O neurons has never been previously described in birds. With the goal of determining whether the chick developmental pattern was more similar to that of mammals or of fish, we investigated the emergence of H/O-expressing cells in the brain of chick embryos of different ages using immunohistochemistry. Post-natal chick brains were included in order to compare the spatial distribution of H/O cells with that of other vertebrates. We found that H/O-expressing cells appear to originate from two separate places in the region of the diencephalic proliferative zone. These developing cells express the H/O neuropeptide at a comparatively early age relative to rodents (already visible at 14% of the way through fetal development), thus bearing a closer resemblance to fish. The H/O-expressing cell population proliferates to a large number of cells by a relatively early embryonic age. As previously suggested, the distribution of H/O neurons is intermediate between that of mammalian and non-mammalian vertebrates. This work suggests that, in addition to its roles in developed brains, the H/O peptide may play an important role in the early embryonic development of non-mammalian vertebrates.

## Introduction

Hypocretins/Orexins (H/Os) are neuropeptides produced by a relatively small number of neurons in the vertebrate hypothalamus (hypocretin 1 and 2 and orexin A and B are independently-coined names that refer to the same peptides) [1], [2]. H/O peptides and their precursors are strongly conserved among higher vertebrates [3], with chicken sequences clustering closely with those of mammals and other tetrapods, while fish sequences are less conserved [4]. The location of H/O neurons in the hypothalamus is also conserved in all vertebrates examined thus far [3]. Mammalian H/O neurons are contained within the dorsal and lateral hypothalamus [5]–[7]. In amphibians, reptiles and fishes, H/O neurons show a more restricted medial periventricular distribution [4], [8]–[10]. In birds, H/O neurons show a distribution pattern that is intermediate between that of mammals and other tetrapods. They are located medially within the paraventricular hypothalamic nucleus, extending into the lateral hypothalamic area [11]–[13] (the terminology used throughout the present paper follows the nomenclature of [14]).

H/O neurons play a critical functional role in the regulation of behavioral arousal and brain state changes in mammals. H/O neurons are generally active during waking and virtually silent during sleep [15]–[17], and promote sleep-to-waking transitions [18]. They also regulate several other physiological functions, stimulating locomotion, feeding, food reward, metabolism, thermogenesis, the neuroendocrine axis, respiration, and the cardiovascular sympathetic and gastrointestinal vagal systems [19]. It has therefore been suggested that H/O neurons act as global activators of a broad range of brain systems [20]–[22]. The conservation of the distribution pattern of H/O fibers also suggests a conservation of H/O function among vertebrates [8]–[10]. H/Os induce arousal in neonatal chickens [23] and larval zebrafish [24], and stimulate feeding and locomotion in fish [4].

Mammalian embryos do not show any signs of a waking-like state being present before birth [25]. Accordingly, they appear to produce H/O peptides late in the gestation period, presumably in preparation for post-natal life. H/O neurons are first detected at about 80-85% of the gestation period in rats [26], [27]. If H/O peptides function primarily after birth, one might expect precocial mammalian species like sheep to start their H/O expression at roughly the same proportion of the way through embryonic development as altricial mammalian species like rats. Sheep fetuses show a good number of H/O neurons and high cerebrospinal fluid levels of H/O by about 85% of gestation [28], suggesting that H/O expression may actually start at earlier embryonic ages in sheep. However, no work to date has directly addressed this question. Fish embryo brains start expressing H/O mRNA between the cleavage and segmentation stages [29]–[31], approximately midway between zygote formation and their larval stage, and it is therefore likely that these peptides are being released at earlier developmental stages than in mammals [32], [33]. Since signs of sleep behavior do not appear until well into the larval stage at about 5 days post-fertilization [24], (an)other role(s) for H/O in fish embryo development is(are) likely. It has previously been suggested that H/Os may be involved “in the complex energy balance during the hatching process” [30]. A direct role in brain embryonic developmental processes also cannot be excluded.

Bird embryos, unlike those of mammals but like those of many fish, are biologically independent from physiological changes that may occur in their mother's body during their entire incubation period. While previous researchers have examined the embryonic development of chicken hypothalamic peptidergic systems [34]–[37], no information has previously been made available about the development of H/O. We therefore investigated H/O expression in the brains of chick embryos using immunohistochemistry. We were particularly interested in whether H/O neurons in bird embryos emerge at relatively earlier developmental stages (as in fish embryos) or later stages (as in mammalian embryos).

Embryos were examined at ages ranging from embryonic day (E) 3 (∼14% of gestation) until the day before hatching (E20, ∼95% of the way through gestation). We extended our neuroanatomical studies to post-natal day (P) 1 and P21 chicks to examine possible differences between pre- and post-natal brains. By E3, the diencephalon is clearly distinguishable in the anterior part of the neural tube [38]. Neurogenesis starts around E4 (∼19% of gestation), when cells abandon the proliferative ventricular zone and form a less dense, post-proliferative mantle zone [39]. Cells then disperse in the mantle zone following migration [40]. Our work thus covers a time span that includes the major neuronal production and migration phases of the chick hypothalamus.

## Methods

### Ethics statement

The experimental protocol for the use of chick embryos was submitted to the McGill University Animal Compliance Office, which, after consultation with the McGill University Animal Care Committee, issued a written waiver stating that according to Canadian and McGill University animal care guidelines, no formal approval was necessary to perform these experiments. McGill Standard Operating Procedures were followed to minimize any possible suffering by embryos. The experimental use and care of P1 and P21 chicks was approved by the McGill University Animal Care Committee.

### Tissue collection

Fertilized chicken eggs (Bovans brown; Couvoir Simetin, Mirabel, QC) were incubated in a commercial egg incubator (Brinsea Octagon, Brinsea Products Inc., Titusville, FL) using constant conditions (37.5°C; 60% relative humidity). Some eggs were incubated until hatching and the chicks were sacrificed within 24 hours after hatching. Brown P21 chicks were commercially obtained (Couvoir Simetin). Embryos were deeply anesthetized by exposing the egg to isoflurane vapors. P1 and P21 chicks were also deeply anesthetized with isoflurane. E3, E4, E5 and E6 embryos were extracted from the egg and immersion fixed in 10% formalin/PBS for 24 hrs at 4°C. Older embryos (E8, E10, E12, E14, E16, E18 and E20) and P1 and P21 chicks were transcardially perfused with Howard Ringer's solution (123 mM NaCl, 1.5 mM, 5 mM KCl; 2 min) and then with 10% formalin/1× chick embryology PBS (150 mM NaCl, 2.8 mM KH_2_PO_4_, 7.2 mM K_2_HPO_4_; 10 min) using a peristaltic pump set at a flow rate appropriate for each age (1–15 mL/min). The heads were kept in 10% formalin/1× chick embryology PBS at 4°C overnight. Brains from E10–E20 embryos and P1 and P21 chicks were extracted from the skull. The basal part of the skull containing the pituitary gland was retained for E12, E16 and E20 embryos.

Whole embryos (ages E3–E6), heads (age E8) and the isolated brains and skull bases (ages E10–E20, P1, P21) were cryoprotected in 10% sucrose/1× chick embryology PBS for 1 day and in 30% sucrose/1× chick embryology PBS for 2–3 days until they sank. They were then frozen at -65°C and cut into 40 µm thick coronal sections at a cryostat (Leica CM3050; Leica Microsystem Canada, Richmond Hill, ON). All sections were collected and deposited on glass slides and kept at −65°C.

We examined the brains from E3–E8 with n = 3 embryos for each age, from E10–E20 with n = 4 embryos for each age and from P1 and P21 with n = 4 and n = 2 chicks, respectively. The pituitary gland was examined at E6 (when it first became distinguishable) with n = 3 embryos, E8 (n = 3), E12 (n = 2), E16 (n = 3) and E20 (n = 4).

### Immunohistochemistry, quantification procedures and statistical analyses

Immunohistochemical staining of every other section was performed following standard protocols. Slides were incubated with blocking solution containing 10% normal horse serum, 2% bovine serum albumine, 0.5% Triton X-100 in 1×PBS (137 mM NaCl, 1.5 mM KCl, 8 mM Na_2_HPO_4_, 2.7 KH_2_PO_4_, pH 7.4; 1 hour at room temperature, RT), a goat polyclonal anti-H1/OA primary antibody (Santa Cruz Biotechnologies, sc-8070; 1∶500 in blocking solution; overnight at RT), a biotinylated secondary antibody (Vector Labs; 1∶500 in blocking solution; 1 hour at RT), the avidin-peroxidase complex (Vector Labs; 1∶200 in 1×PBS containing also 0.5% Triton X-100; 1 hour at RT) and 3,3′-diaminobenzidine (Sigma) as a revelation system. Omission of the primary antibody or preincubation of the primary antibody with the blocking peptide (Santa Cruz Biotechnologies, sc-8070P; following company protocol) did not yield any labeling. The primary antibody also recognizes H2/OB but does not recognize the precursor protein (Technical Service, Santa Cruz Biotechnology, Inc.; personal communication and antibody datasheet).

All quantification procedures were conducted blindly with reference to the source of the sections. The total number of labeled cells was estimated using a Leica DM6000 B microscope with the 20X objective lens and the computer-assisted optical fractionator method of stereology using Stereo Investigator (Version 10.04.2, MicroBrightField, Inc., Williston, VT). Within each brain section, contours were traced around clusters of labeled cells in the hypothalamus. Each contour was quantified in its entirety using side by side counting frames. Following set inclusion and exclusion boundaries on each counting frame reduced the possibility of counting the same cell twice. Sections that were damaged or lost were considered missing. The total number of cells was then extrapolated taking into account the sections that were not labeled. Kruskal-Wallis ANOVAs with posthoc tests corrected for multiple comparisons [41] were used to evaluate the significance of differences among mean cell number values at the various developmental stages.

The cranio-caudal extension of the H/O hypothalamic area was estimated by multiplying the total number of sections containing H/O neurons by 2 (we labeled every other slide) and by the 40 µm section thickness. Kruskal-Wallis ANOVAs with posthoc tests corrected for multiple comparisons [41] were used to evaluate the statistical significance of differences among mean extension values at the various developmental stages.

### Antibody specificity

The supplier has not validated the primary antibody used in this study (sc-8070) by Western blot in any animal species. It has been used for immunohistochemical studies in both mammalian and non-mammalian species. In some of these species, the antibody specificity was established by comparison with another antibody of known specificity [42]. In other cases, the immunostaining was accompanied by a Western blot using an antibody against prepro-H/O [43]. In one case, the antibody was reported to have “passed the Western blotting test” but details were not provided about the test [44]. The low molecular weight and low expression levels of the H/O peptides makes their visualization with Western blot challenging. We are not aware of any other published Western blot analysis using the same antibody we used here (sc-8070) in any animal species. Using a different antibody from ours, H1/OA was detectable in the hypothalamus of transgenic mice overexpressing prepro-H/O, while no band was seen in wild-type mice [45].

The specificity of the antibody used here in chicken embryo brains is suggested by the sequence analysis described below. We were also able to demonstrate it directly by Western blot analysis [46].

#### Sequence analysis

The anti-H1/OA primary antibody was raised against a 19 residue peptide mapping at the C-terminus of the human H1/OA (aminoacids 48–66 of the human precursor protein). The immunogen shares 84% sequence identity with both H1/OA and H2/OB of *Gallus gallus* origin (protein accession # NP_989516.1). The immunogen sequence was entered into DELTA-BLAST [47] and the only sequence in the *Gallus gallus* proteome producing significant alignments is that of prepro-H/O (protein accession # NP_989516.1), at the level of amino acids 43–61 (E value 2e-9; corresponding to the C-terminus of the H1/OA sequence) and 74–91 (E value 1e-8; corresponding to the C-terminus of the H2/OB sequence). This analysis suggests that the sequence recognized by the antibody is very specific to the two H/O peptides.

#### Western blot analysis

Fertilized chicken eggs (Bovans brown; Couvoir Simetin, Mirabel, QC) were incubated as above. E20 embryos in the egg (n = 8) were anesthetized with isoflurane, extracted from the egg and decapitated. The brains were quickly extracted and frozen at −75°C. The hypothalamus was dissected from the frozen brain on a cryostat in two different ways: a) the whole posterior hypothalamus (n = 4); b) a more precise dissection of the posterior hypothalamus excluding areas in its dorsolateral part and in its more posterior parts which do not contain H/O cells (n = 4). Brain sections were cut just before and after dissection, fixed and then stained with Cresyl Violet to confirm the proper dissection of the hypothalamus.

Each dissected hypothalamus was homogenized in ice-cold HEPES-buffered sucrose buffer (20 mM HEPES, 320 mM Sucrose, 1.0 mM EDTA, 2.0 mM EGTA, pH 7.4) supplemented with protease inhibitor cocktail (Pierce). Synaptosomes were prepared by a sucrose-density gradient centrifugation method [48]. Protein concentration from total homogenate and synaptosomal fraction was determined by a bicinchonic acid assay (Pierce). Samples (40 µg of total protein/lane) were mixed with 2X loading Sample buffer (200 mM Tris-HCl pH 6.8, 40% glycerol, 2% SDS, 0.04% Coomasie blue, 2% β-mercaptoethanol), incubated at 37°C for 15 min and loaded on a Tricine 10–20% gel (BioRad) for SDS-PAGE with Tris-Tricine SDS running buffer [49]. Blocking peptide solutions (sc-8070P; 0.1 µg/lane) were treated with the same protocol and loaded on separate lanes. Proteins were transferred onto PVDF membranes (0.2 uM pore size, Millipore) at 4°C and at 20 V for 18 h. Membranes were blocked with 3% BSA in Tris-buffered saline containing 0.1% Tween 20 (TBS-T) and incubated with the anti-H1/OA antibody (sc-8070; 1∶500) in TBS-T for 1 hour at RT. After 3 washes in TBS-T, the membranes were incubated with HRP-conjugated anti-goat secondary antibody (Millipore; 1∶5000) in TBS-T for 1 h at RT, followed by 3 washes in TBS-T. Membranes were treated with enhanced chemiluminiscence (ECL) Plus Western blotting substrate (Pierce) or SuperSignal West Femto Chemiluminescent Substrate (Pierce) and subsequently exposed to autoradiography film or scanned with a Storm Laser Scanner (Molecular Dynamics).

We inconsistently detected a faint band at ∼3 kDa corresponding to the processed H/O peptides in the fresh synaptosomal fraction, but never in the total homogenate obtained from dissected whole hypothalamus. H/O peptide concentration in the adult rat hypothalamus is about 10^−6^ g/g of total proteins [50]. We loaded 40 µg of total hypothalamic protein/lane and we were not able to detect a consistent H/O band using a sensitive revelation system that has a detection limit of 40 pg of protein/lane. This suggests that H/O levels in the chick E20 posterior hypothalamus are lower than in the adult rat and below the detection limit for Western blot analysis.

**Figure 1:**
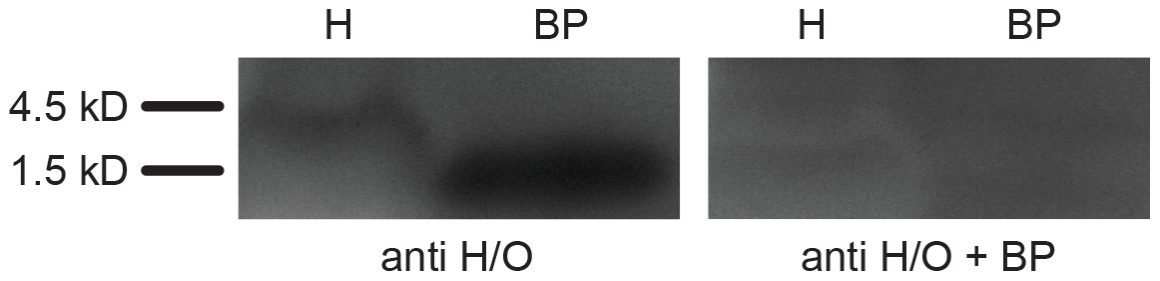
Western blot analysis of the anti-H1/OA antibody in the chick embryo hypothalamus. Synaptosomal preparations from the dissected hypothalamus from an E20 chick embryo (H; 40 µg of proteins) and the blocking peptide (BP; 10 µg) were loaded. The anti-H1/OA antibody binds to a hypothalamic peptide (∼3 kDa) and to the blocking peptide (∼1.5–2 kDa) (anti H/O; left panel). Preincubation of the anti H1/OA antibody with the blocking peptide does not yield any band (anti H/O + BP; right panel).

With the more precise dissection of the hypothalamus, we were able to reliably obtain a band at ∼3 kDa (Figure 1, left panel). The antibody did not recognize any other protein from the tissue extracts including the chicken pro-H/O, in agreement with the antibody data sheet and information provided (Santa Cruz Biotechnology, Inc. Technical Service). It recognized the 19 amino-acid blocking peptide as a ∼1.5–2 kDa band (Figure 1, left panel). Preincubation of the primary antibody with the blocking peptide (sc-8070P; following company protocol) did not yield any band (Figure 1, right panel).

## Results

Neuronal and glial precursors and differentiating cells can be distinguished during development based on ultrastructural features observed with electron microscopy [51], or by the expression of selective markers [52]. Until E4-E5, cells in the proliferative ventricular zone are still capable of division and considered undifferentiated [51]. Once they stop dividing, cells leave the ventricular zone and move to the developing mantle zone where they undergo migration and differentiation [39]. Since we did not perform either one of the above-mentioned analyses to assess the developmental status of H/O-labeled cells, we refer to them as “cells” at earlier ages. Starting from E10 inclusive, we call them “neurons” because of the presence of H/O-positive varicose terminals (axons) in the hypothalamus.

### The overall developmental pattern of H/O cells

**Figure 2:**
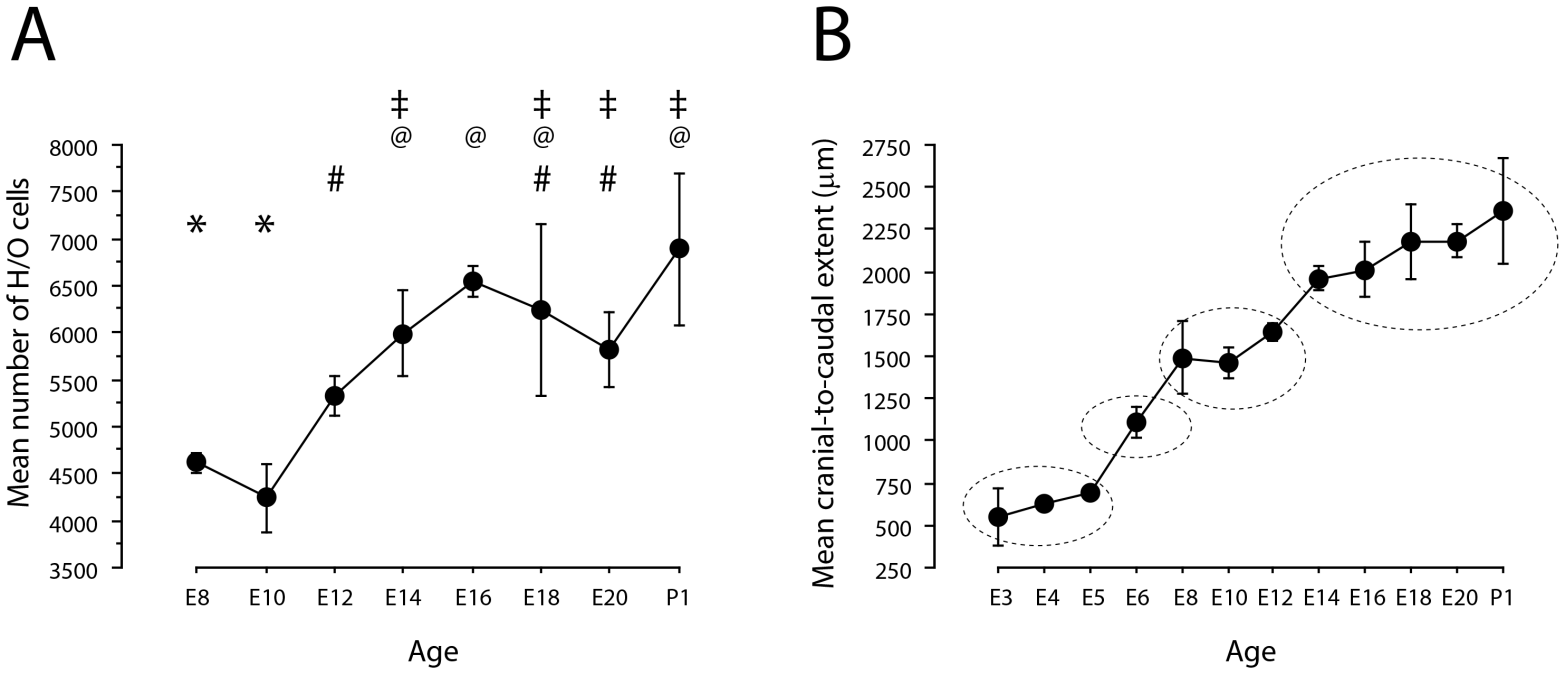
Mean number of H/O neurons (A) and their cranio-caudal spatial extent (in µm) (B) in the brains of embryos at different ages. Bars represent ± 1 SEM and n = 4 for all ages, except for incubation periods of 8 days or less, where n = 3. For each graph, points that do not share symbols above them are significantly different from each other at the *p*<0.05 level, two-tailed, corrected for multiple comparisons (Kruskal-Wallis ANOVA; H = 15.96, df = 6, *p* = 0.014 in A; H = 33.05, df = 10, *p* = 0.0003 in B).

Cells start producing H/O peptide at extremely early ages in chicken embryos. H/O cells were already detectable by E3, the youngest age we examined. We counted ∼1000 labelled cells at this age (14% of P1 values; Table 1). We were not able to reliably count H/O cells at the ages of E4-E6, because labeled cells became very densely packed and often indistinguishable as independent entities using light microscopy in our immersion fixed preparations. We systematically quantified labeled cell numbers starting from E8 (Table 1, Figure 2, A), the youngest age at which embryos were perfused. The estimated number of H/O cells was already high at E8 and E10 (∼4000–4500; 62–67% of P1 values), with a significant increase to ∼5500 (77% of P1 values; E12), ∼6000 (87% of P1 values; E14), ∼6500 (95% of P1 values; E16) by about 3/4 of the way through the incubation period. Values were still high at ∼6200 at E18 (91% of P1 values). A decrease (significant with respect to E16) was then seen at E20 (∼5800 H/O cells; 85% of P1 values) while the value at P1 reached ∼6900 H/O cells. The cranio-caudal spatial extent of the hypothalamic area containing H/O cells was also estimated for all ages (Table 1; Figure 2, B). At E3 it was about 0.5 mm, significantly increasing over the period including E6 and E8, when a value of about 1.5 mm was obtained. A significant increase was later seen at E14, when a plateau was reached at a value of about 2 mm. (Our examination of the P21 chicks was limited to the description of H/O neuronal distribution; no H/O neuron counts were performed at this later age).

**Table 1:** Total number (mean per animal) of H/O positive cells found in the entire hypothalamus, and cranio-caudal spatial extent (in µm) of the hypothalamic area containing H/O cells at different ages.

Age	n	Total number of H/O cells (mean ± SEM)	Cranio-caudal extent (µm; mean ± SEM)
E3	3	964 ± 417	547 ± 170
E4	3	nc	627 ± 35
E5	3	nc	693 ± 35
E6	3	nc	1107 ± 93
E8	3	4614 ± 114	1493 ± 218
E10	4	4242 ± 359	1460 ± 89
E12	4	5333 ± 217	1640 ± 52
E14	4	5994 ± 450	1960 ± 69
E16	4	6544 ± 164	2010 ± 162
E18	4	6236 ± 913	2180 ± 222
E20	4	5825 ± 392	2180 ± 100
P1	4	6889 ± 812	2360 ± 314

n: number of animals; nc: not collected; SEM: Standard Error of the Mean.

### From E3-E6, cells begin expressing H/O peptides before they migrate from the ventral proliferative zone, and change the orientation of their neurites as they migrate laterally

**Figure 3:**
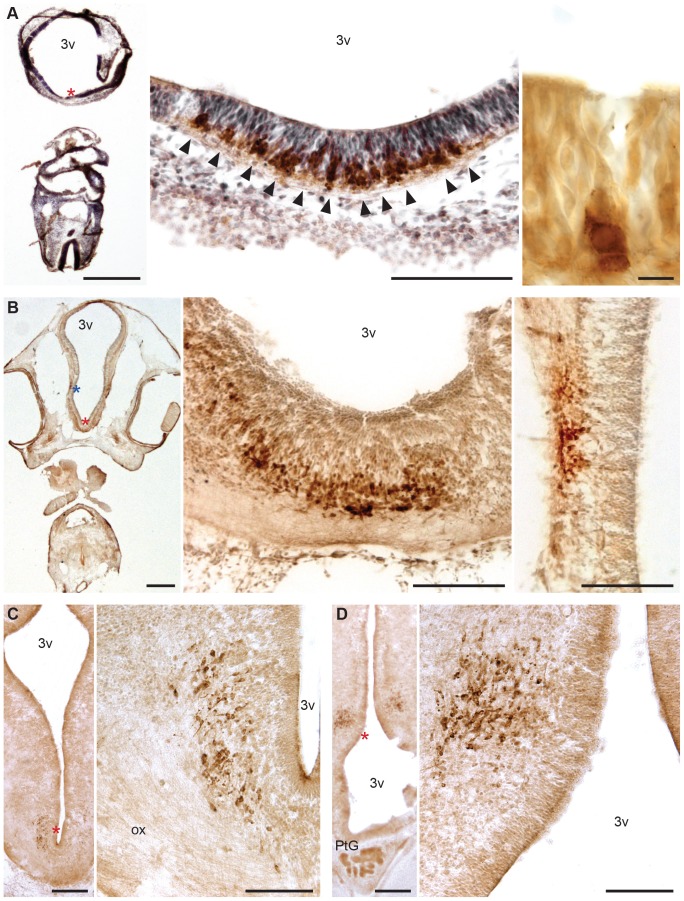
H/O cells in chick embryos at earlier ages (E3–E6). H/O cells appear brown. In A, the left panel shows a low-magnification view of an E3 embryo coronal section stained for H/O and counterstained with Cresyl Violet. The star indicates the ventral part of the diencephalic vesicle that is shown at higher magnification in the middle panel. H/O cells appear grouped in clusters (arrows). The right panel show one of these clusters of labelled cells at higher magnification in a consecutive section that was not counterstained with Cresyl Violet. In B, the left panel shows a low-magnification view of an E4 embryo coronal section stained for H/O. The stars indicate two locations in the ventral part of the diencephalic vesicle that are shown at higher magnification in the central (red star) and right (blue star) panels. In C and D, the left panels show a low-magnification view of an E6 embryo coronal section stained for H/O at two different levels. At more anterior levels (C), the star indicates a labeled ventral region that is shown at higher magnification in the right panel. At more posterior levels (D), the star indicates a labeled region in a more dorsal location, shown at higher magnification in the right panel. 3v: 3^rd^ ventricle; ox: optic chiasm; PtG: pituitary gland. Scale bars are 500 µm (A–D, left panels), 10 µm (A, right panel), and 100 µm (A–D, other panels).

At **E3**, H/O cells were seen at medial locations in the ventral part of the ventricular zone of the diencephalon and distributed in clusters (Figure 3, A). The cranio-caudal level corresponds to that of the optic stalk. Labeled cells were seen in the outer third/half of the thickness of the ventricular zone. They were round in shape with a big nucleus surrounded by a thin and unevenly labeled cytosol. Some of these cells also showed short labeled neurites that ran towards the outer surface of the neural tube. From here, these labeled neurites were occasionally seen turning in a laterodorsal direction. At **E4** and **E5**, labeled cells were seen concentrated in two discrete groups in the diencephalic wall, one along the ventral midline and the other more dorsally at about 1/3 of the total ventrodorsal extension of the diencephalic wall (Figure 3, B). H/O cells had a roundish shape, were more strongly labeled than at E3, and had short, strongly-labeled neurites. Cranially, H/O cells first appeared at the level of the optic stalk (levels d, c and b of plate 84 in [38]). They were located in the outer third/half of the diencephalic wall at more anterior and ventral levels with mostly radially-oriented neurites. More posteriorly and more dorsally, a few labeled cells were seen in the diencephalic wall with radially-oriented neurites (Figure 3, B). Most H/O cells were seen just outside the ventricular zone of the diencephalon intermingled with fibers and unlabeled cells in the developing mantle zone. The orientation of their labeled neurites changed from strictly radial (closest to the diencephalic wall) to a variety of directions (more laterally) (Figure 3, B).

By **E6**, H/O cells were still roundish in shape and their neurites were longer than at younger ages (except at the most cranial and medial levels). Starting cranially at the level of the optic nerves, strongly labeled cells were seen along the midline in the ventral part of the diencephalic wall, between the ventricular zone and a thick fiber layer corresponding to the developing optic chiasm (Figure 3, C). Their neurites had a radial orientation. More caudally, H/O cells were not strongly labeled and were present more dorsally and bilaterally in the thicker mantle zone of the ventral part of the diencephalon. Their neurites had a radial orientation. At the caudalmost levels, a patch of strongly labeled H/O cells was seen bilaterally in the diencephalic wall, above the third ventricle enlargement (Figure 3, D). The medial-most cells (closer to the proliferative zone) were generally less strongly labeled than the more lateral cells, and their labeled neurites had a radial orientation. The more laterally-located cells had neurites extending in diverse directions.

### From E8-E20, H/O cells populate the developing hypothalamus

At **E8**, the optic chiasm becomes prominent and marks the most cranial location at which H/O cells were seen. At this level, labeled cells were seen around the ventral tip of the 3^rd^ ventricle, just outside the densely packed circumventricular proliferative zone. A few cells were seen more dorsally and bilaterally, along the ventral half of the 3^rd^ ventricle. These cells had neurites that were oriented in a ventro-dorsal direction. About 200–300 µm more caudally, a bigger concentration of H/O cells was seen bilaterally half-way up the 3^rd^ ventricle with neurites oriented in different directions. A few cells were present also in ventrolateral locations with neurites oriented in a dorsomedial-to-ventrolateral direction. H/O cells were rather roundish and not very strongly labeled at these cranial-most levels. More caudally, H/O cells were still seen in great numbers bilaterally next to the 3^rd^ ventricle about half way up, with neurites oriented in different directions. A great number of labeled cells were also seen extending in ventrolateral directions with neurites oriented in dorsomedial-to-ventrolateral directions. More strongly-labeled cells with elongated shapes and strongly-labeled neurites were seen at these more caudal and ventrolateral locations.

**Figure 4:**
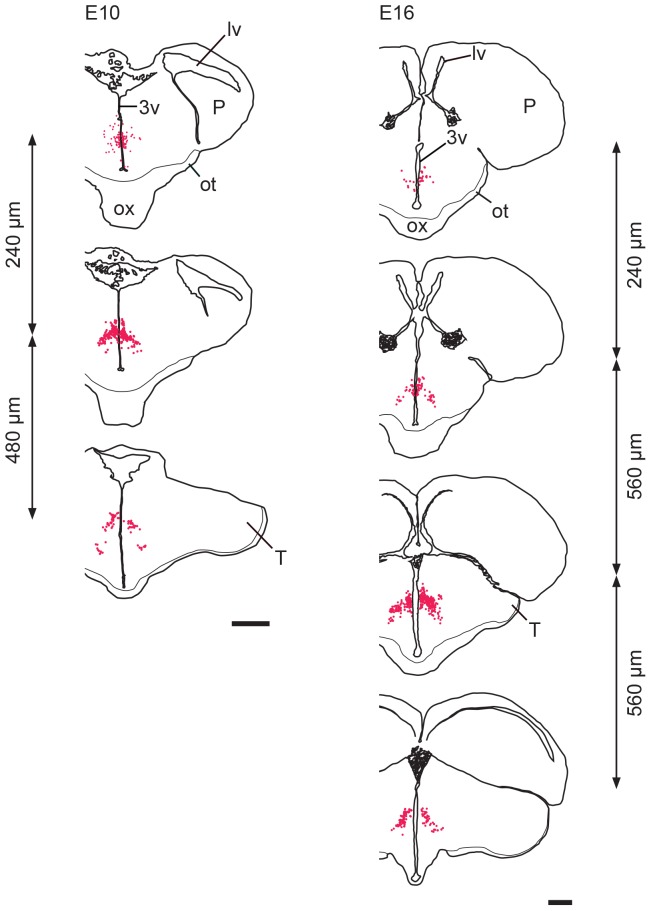
Distribution of H/O neurons (red dots) in the hypothalamus of E10 (left column) and E16 chick embryos (right column). The most cranial level is shown on top and the most caudal one on the bottom. The distance between consecutive levels is indicated on the side. 3v: 3^rd^ ventricle; lv: lateral ventricle; ot: optic tract; ox: optic chiasm; P: pallium; T: tectum. Scale bars are 1 mm.

**Figure 5:**
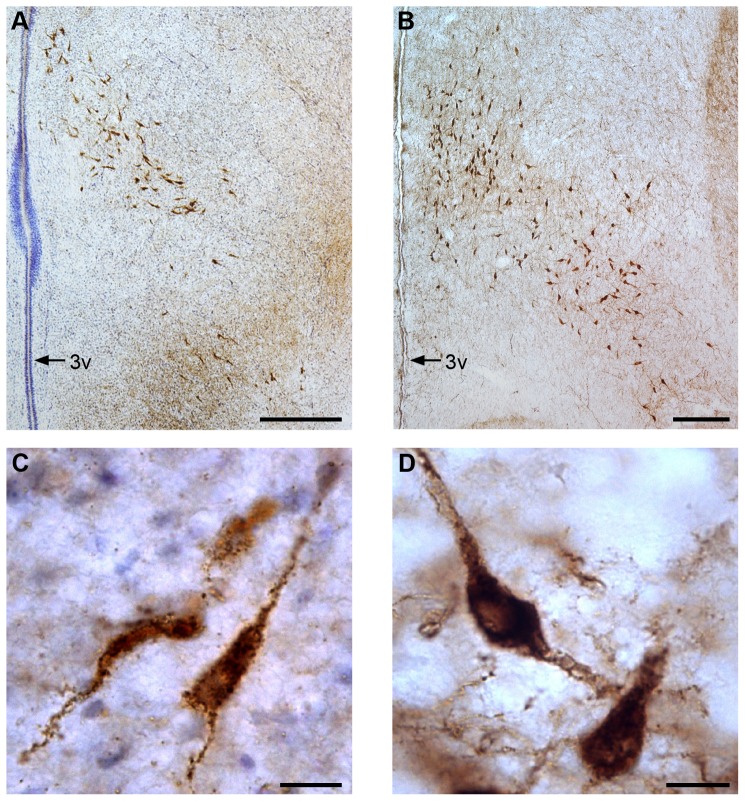
H/O neurons in E10 and E20 chicken embryos. H/O neurons appear brown. Coronal sections of the caudal portions of the hypothalamus show the distribution of labeled neurons in a representative E10 embryo at low (A) and at high magnification (C) and in a representative E20 embryo at low (B) and at high magnification (D). The sections from the E10 embryo were counterstained with Cresyl Violet to better identify the brain structures. The inverted V-shape distribution of H/O neurons is evident at both ages. Labeled neurons appear bigger and more strongly labeled at E20 than E10. 3v: 3^rd^ ventricle. Scale bars are 200 µm (A, B) and 10 µm (C, D).

By **E10** and **E12**, a small number of labeled neurons remained close to the circumventricular proliferative zones (Figure 4, top panel). A few labeled neurons were found scattered bilaterally along the 3^rd^ ventricle; these were more concentrated at more dorsal levels (Figure 4, top two panels), with neurites oriented in different directions. At E12, these neurons were much more abundant than at E10. More caudally, labeled neurons also extended in a ventrolateral direction, with neurites oriented in dorsomedial-to-ventrolateral directions. The area containing labeled neurons had an inverted V shape, with the vertex located in the middle third of the 3^rd^ ventricle (Figures 4, bottom panel and 5, A). H/O neurons showed an elongated shape with strongly-labeled cell bodies and neurites (Figure 5, C). H/O-positive varicose terminals were first seen in the hypothalamus at E10.

At **E14**, the hippocampal (pallial) commissure became visible above the 3^rd^ ventricle. Starting from this age, H/O neurons were found starting just posterior to it. H/O neurons showed multiple labeled neurites, and were bigger and rounder than at earlier ages, especially in ventrolateral and posterior locations. Cranial H/O neurons were seen in small numbers mostly in a medial position lining the ventral half of the 3^rd^ ventricle at **E16** (Figure 4, first panel from the top) and **E18**. Neurons here had their main neurites oriented in a dorsoventral direction. At more caudal levels, a high concentration of labeled neurons was seen lateral to the 3^rd^ ventricle at **E16** (Figure 4, second and third panels from the top) and **E18**. These neurons had their main neurites oriented in various directions. From here, H/O neurons also extended in a ventrolateral direction, filling an area with an inverted V shape (Figure 4, second-fourth panels from the top), with neurites oriented in the same direction.

**Figure 6:**
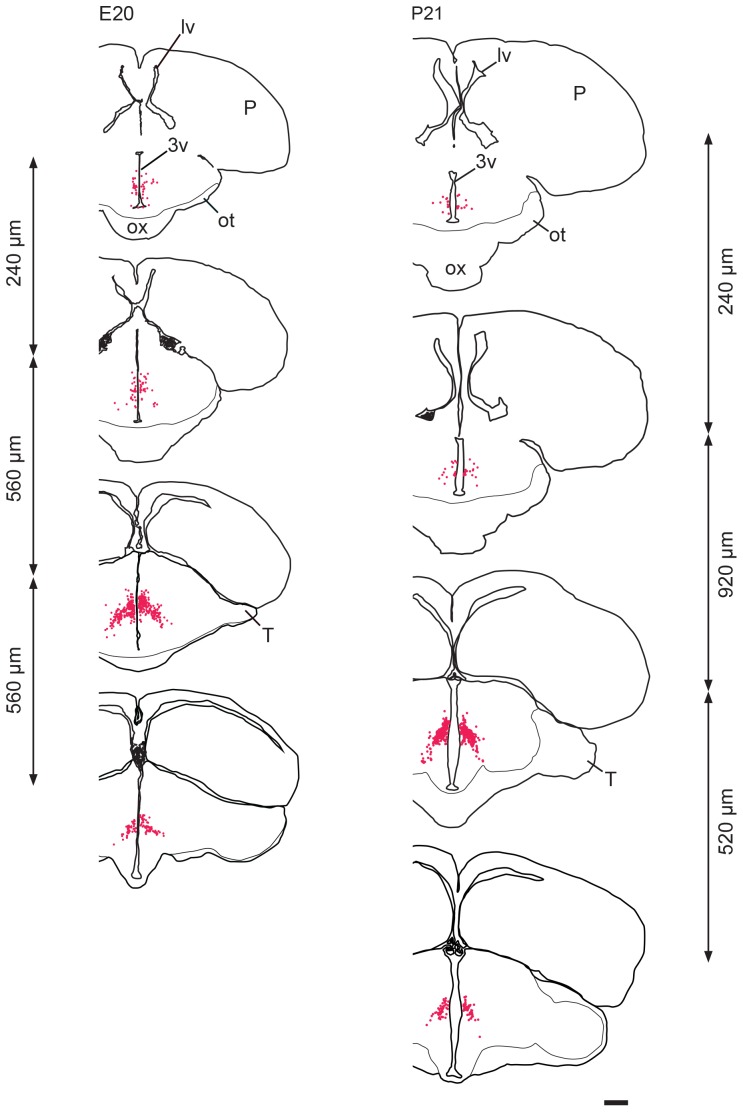
Distribution of H/O neurons (red dots) in the hypothalamus of E20 chick embryos (left column) and P21 chicks (right column). The most cranial level is shown on top and the most caudal one on the bottom. The distance between consecutive levels is indicated on the side. 3v: 3^rd^ ventricle; lv: lateral ventricle; ot: optic tract; ox: optic chiasm; P: pallium; T: tectum. Scale bar is the same for both columns and is 1 mm.

At **E20**, a few labelled cells were still seen around the ventral end of the 3^rd^ ventricle (Figure 6, top panel). There appeared to be more H/O neurons than at earlier ages located at the most anterior levels, lining the ventral half of the 3^rd^ ventricle, corresponding to the developing rostral part of the hypothalamic paraventricular nucleus (Figure 6, top two panels). At more posterior levels, H/O neurons were seen in regions corresponding to the magnocellular and parvocellular parts of the developing paraventricular nucleus, and in the periventricular stratum. From here, labeled neurons extended ventrolaterally into an area that included the developing posterior hypothalamic nucleus and lateral hypothalamic area, filling an area with an inverted V shape (Figures 5, B and 6, bottom two panels). There also appeared to be more H/O neurons in ventrolateral locations at E20 than at earlier ages. H/O neurons showed a more roundish shape and a stronger labeling than at E10 (Figure 5, D).

### At P1 and P21, the spatial distribution of H/O neurons is unchanged with respect to E20

At **P1**, the spatial distribution of H/O neurons was similar to what was described at E20, including a few labelled cells around the ventral end of the 3^rd^ ventricle.

At **P21**, brain size was substantially increased with respect to E20 and P1, but the spatial distribution of H/O neurons in the posterior hypothalamus was the same. At more anterior levels, labelled neurons were found in a medial location close to the 3^rd^ ventricle in the paraventricular hypothalamic nucleus and in the periventricular stratum (Figure 6, top two panels). At more posterior levels, labelled neurons were distributed in ventrolateral locations corresponding with the posterior hypothalamic nucleus and lateral hypothalamic area, filling an area with an inverted V shape (Figure 6, bottom two panels). No H/O neurons were detected around the ventral end of the 3^rd^ ventricle.

### H/O labeling did not enter the developing pituitary gland

A few H/O labeled cells were seen in the infundibulum at E6. This is not surprising, since this structure is continuous with the one lining the ventral part of the 3^rd^ ventricle, where H/O neurons appear to come from. At E6, a few labeled cells were also seen in the epithelium of the distal part of Rathke's pouch, in close proximity to the developing pituitary gland. Despite a careful search, we could not detect any labeled fibers in the pituitary gland at any of the ages we examined. Well-stained H/O varicose fibers were seen in the infundibulum at E12, E16 and E20.

## Discussion

Using an anti-H1/OA antibody whose molecular specificity was confirmed with Western blot analysis, we have quantified the development of H/O-positive cells in the brain of chick embryos. In agreement with the present results, H/O neurons have previously been described in post-natal chickens at similar locations using in-situ hybridization [11],[53] (compare Figure 6, bottom two panels with Figure 3 from [11] and Figure 1 from [53]).

By E3, five regions can be distinguished in the anterior part of the neural tube (telencephalon, diencephalon, mesencephalon, metencephalon and myelencephalon), the telencephalic vesicles are developing, and the cranial and cervical flexures have appeared in the chick embryo [38]. Neurogenesis then starts around E4 (∼19% of gestation), when cells abandon the proliferative ventricular zone and form a less dense, post-proliferative mantle zone [39]. Cells then disperse in the mantle zone following radial and tangential migration [40]. Hypothalamic neurons are generated mostly from the ventricular zone lining the third ventricle in the embryonic diencephalon [54]. The hypothalamus is generated with an outside-inside pattern, with three successive but overlapping waves of neurons giving origin to the lateral/reticular, the core and the midline hypothalamus, respectively [54]. Previous work on the neuroendocrine system in the posterior hypothalamus of chick embryos has described how different types of parvocellular and magnocellular neurons are first detected starting around E5 [55]. The present work thus covers a time span that includes the major neuronal production for and migration into the chick hypothalamus.

The main result from the present work is that H/O cells appear to be the first neurochemically distinct population to become detectable in the developing chick embryo hypothalamus (by E3 or earlier). These cells start producing H/O when they are still in the ventricular zone.

### Chicken H/O neurons appear to originate from 2 different locations in the diencephalic proliferative zone

Chicken H/O cells are already detectable by E3 in the external part of the ventricular zone of the 3^rd^ ventricle. Previous work has shown that the initial migration of neurons from this proliferative zone is strictly radial, with non-radial migration seen only outside of the ventricular zone starting from E5 in the developing chick diencephalon [56]. The location of the labeled cells at E3–E4 should therefore indicate their site of origin in the proliferating diencephalic wall. It appears that there are 2 separate locations in the proliferative epithelium lining the 3^rd^ ventricle where H/O cells originate: an anterior/ventral/medial location and a more posterior/dorsal/bilateral location. Based upon the position and morphology of labeled cells around these proliferative zones, the ventromedial zone seems to still generate H/O cells until at least P1. The present results cannot exclude the possibility that they may also generate cells (perhaps for a more prolonged period of time) that do not start expressing H/O peptide until later on, as seen in rats [26], [27], [57]. This early start of H/O production in the chick diencephalon should facilitate the identification of possible H/O-neuronal fate determinants.

### Migrating H/O cells appear to follow two different migratory paths

Since this study used only coronal brain sections, it is only possible to make inferences about the migration of H/O cells in the dorsoventral direction (observable within sections) but not in the cranio-caudal direction (going between sections). Previous observations suggest that neurites of developing hypothalamic neurons appear “at the time and place of non-radial migration” [56], and that cells migrate along the direction assumed by their cellular processes [56]. The orientation of the neurites should therefore indicate the direction of cell migration. Chicken embryo H/O cells originating from the ventromedial proliferative zone thus appear to migrate dorsally along the 3^rd^ ventricle; these may represent the main source of the most medial (and anterior) group of H/O neurons. These H/O neurons may also reach more dorsal positions and contribute to the intermediate dorsal concentration of H/O neurons, together with the neurons originating from the more dorsal proliferative zone. H/O cells in this intermediate dorsal group show neurites with different orientations, some in the dorsoventral direction and others in a mediolateral direction (within a single section). H/O cells originating from the more dorsal proliferative zone might instead represent the main source of the more caudal neurons concentrated in the ventrolateral locations in the hypothalamus. Since the ventromedial proliferative zone seems to produce H/O cells until later developmental times, the consequence is that H/O neurons in the intermediate dorsal position (corresponding in part to the paraventricular hypothalamic nucleus) would be generated later than those in the ventrolateral position (corresponding to the lateral hypothalamic area). This would be in accordance with what has been observed in rats [54].

Interestingly, the two patterns of migrations proposed here for H/O cells (in a ventral-to-dorsal direction along the 3^rd^ ventricle and in a dorsomedial-to-ventrolateral direction) have been described for cells giving rise to other hypothalamic areas in mice [58]. An alternative hypothesis is that H/O cells could migrate just laterally from their site of origin in the ventromedial proliferative zone and reach the ventrolateral locations. This hypothesis is contradicted by the complete lack of H/O cells showing neurites oriented in mediolateral directions within this area or present in intermediate positions between the ventromedial proliferative zone and ventrolateral positions. However, in order for the migratory pathway of H/O cells to be established with certainty, further experiments using specific neuronal markers for tracking cell movements in vivo will be necessary. Early expression of H/O provides a tool for the identification of this cell population in chick embryos.

### Chickens appear to produce more H/O neurons at earlier times in embryonic development than rats do

The present experiments showed that chicken H/O neurons express peptide from the earliest ages examined (E3, 14% of the embryonic period) and that the number of H/O neurons increases during chick embryonic development up to a maximal value of about 6500 around age E16 (about 75% of the fetal period). A similar number of H/O cells were seen at P1. At E20, we observed a small decrease in H/O cells which was significant with respect to E16 (but not to E18 or P1). Further experiments are needed in order to ascertain whether cell death could contribute to this phenomenon.

Among mammals, the development of H/O neurons has been mostly studied in rats; the data vary among studies. Wistar rats appear to reach a maximum of ∼2500 H/O neurons during the 9^th^–10^th^ postnatal week in one study [59] and to have ∼6700 neurons in another study (rats of 200–250 g) [60]. Values of 3500–4000 have been reported for “adult” Sprague-Dawley rats [61], an unidentified strain [62] and Flinders Sensitive and Flinders Resistant Line rats [63]. Most of these values are notably lower than those we obtained in chick embryos, suggesting that H/O neurons may play a more prominent role in the development of bird embryos with respect to mammalian embryos (see below).

In rats, H/O neurons are generated in one sharp peak around E12 [64] (∼55% of gestation) but they only become detectable by immunohistochemistry starting at E18 (∼82% of gestation) using an anti-prepro-peptide antibody [27], or E19 (∼86% of gestation) using an anti-H2/OB antibody [26], or after P10 using an anti-H1/OA antibody [57]. At these stages the H/O neurons are already well-developed, as demonstrated by the contemporaneous detection of labelled fibers in the hypothalamus and locus coeruleus [26], [27]. While an exact determination of when the H/O neurons that function in the developed brain are generated in chick embryos requires birthdating experiments, H/O peptides are clearly being produced at earlier ages than in rats.

### Comparison with the development of H/O neurons in non-rodent mammalian and non-mammalian species

As mentioned in the Introduction, sheep fetuses show a sizeable number of H/O neurons by about 85% of gestation [28]. The presence of high cerebrospinal fluid levels of H/O at the same age suggests that H/O expression actually starts at earlier embryonic ages in sheep; however, no work to date has addressed this question. Similarly, in another relatively precocial species, the pig, prepro-H/O mRNA is detectable in fetuses at 93% of gestation but no younger ages were examined. More research is needed into the development of H/O neurons in precocial mammals, like sheep and guinea pigs, to understand whether H/O expression in embryos varies as a function of developmental pattern.

Although H/O expression was first found here at the segmentation stage in chick embryos, in fish it has been reported to start earlier, in stages ranging from the cleavage stage in the Atlantic cod [31], to the neurula stage in the orange-spotted grouper [30] and the segmentation stage in zebrafish [29]. Future work is needed to demonstrate how early H/O expression actually starts in chickens and other birds.

### H/O neurons in chickens are distributed differently within the hypothalamus than in other animal species

In adult vertebrates, H/O neurons are mostly found in the hypothalamus, but differences occur among different species. In chickens, the majority of H/O neurons are distributed in a dorsomedial position in the paraventricular and posterior hypothalamic nuclei. A lower density of H/O neurons is found extending in a ventrolateral position (into the lateral hypothalamic area). A smaller but significant number of neurons is present in ventromedial positions lining the 3^rd^ ventricle, especially at more anterior levels of the posterior hypothalamus. This distribution is consistent with the one previously described in chickens [11] and other birds [12], [13].

In reptiles, amphibians and fish, hypothalamic H/O neurons are primarily arranged in medial locations, lining the 3^rd^ ventricle, in both the anterior and posterior portions [4], [9], [10]. Fish deviate from other vertebrates in having major extra-hypothalamic populations of H/O neurons [65]. In most mammals, H/O neurons are concentrated in the lateral hypothalamus and are lacking in medial periventricular locations [1], [66]. Our observations lend support to the previously-stated idea that the avian anatomical H/O pattern may be intermediate between that of amphibians and mammals [11]. Like reptiles, amphibians and fish, chickens present a medial periventricular H/O neuronal population. Like mammals, chickens present H/O neurons in dorsomedial and ventrolateral locations, although they are missing a dorsolateral extension. How differences in anatomical location may be reflected in the functional organization of chicken H/O neurons requires further investigation.

### What functions might the H/O peptides serve in embryos?

Previous studies have strongly suggested that the adult function of H/O peptides is similar in birds and mammals. In fact, intracerebroventricular administration of H1/OA increases arousal [23], possibly by activating the monoaminergic systems in chickens [67]. This makes the differing embryonic patterns in the expression of this peptide all the more interesting.

Since the antibody used in this work appears to recognize only the active H/O peptides, the present results suggest that H/O may be released during very early developmental stages in chick embryos. There are some preliminary indications that H/O may play a role in brain development in higher vertebrates. H/O peptides seem to promote synaptogenesis and neuronal maturation [68], [69] as well as neuronal survival [70]. Early expression and release of H/O may thus facilitate the precocial development of circuits underlying all different functions regulated by H/O [19], including circuits involved in eventually maintaining a waking state.

If this is the case, one might expect that H/O would be produced earlier in precocial than in altricial species, since they require such circuits to mature more rapidly. A confirmation or refutation of this hypothesis in precocial (like the sheep) and altricial (like the rat) mammals also awaits further experiments.

### Possible role(s) of H/O in the regulation of metabolism and body temperature

While chicks are precocial at birth [71], the precocity of H/O expression observed here in embryos appears rather extreme, more akin to the pattern seen in fish [32], where, in adults, H/O has been linked to arousal state regulation, locomotion, foraging/reward-seeking behavior, feeding, and sexual behavior [4], [65]. The most likely intersection of these traits with bird embryos may be in the domains of controlling food absorption from the yolk (feeding) and modulation of spontaneous embryonic movements (locomotion and body temperature control).

One possible role in development is based on the involvement of H/O in energy homeostasis [72]. In adult rats, H/O neurons act as sensors for relevant metabolites and hormones, thus integrating information about nutritional status [19]. They then trigger appropriate physiological responses (arousal, locomotion and food intake) [19], [73]. H/O increases nutrient uptake and utilization in muscle and adipose tissue, and some of these effects may be mediated directly [74]. H/O also increases energy expenditure and body temperature. In adult birds, H/O may regulate energy balance under extreme conditions [75].

H/O could be controlling physiological functions essential for chick (and other non-mammalian) embryos related to obtaining nutrients from stored yolk and to their utilization. These effects may be indirectly mediated through the autonomic nervous system and neuroendocrine axes. H/O could reach the pituitary and control the production of pituitary hormones [76]. Once in the hypothalamo-hypophyseal portal system, H/O could also affect peripheral tissues directly. H/O receptors are expressed in the pituitary and peripheral organs in adult chickens [77]. It would be especially interesting to know whether they are also expressed in the embryonic yolk sac membrane.

In birds, heat production starts prenatally, and is essential for the transition from ectothermy to endothermy [78]. Heat is generated through non-shivering thermogenesis in muscle and liver (birds do not have brown adipose tissue) and is controlled by thyroid hormones [78]. Interestingly, TRH and thyroid hormones are produced early in chick embryos [55], [79]. While H/O plays an important role in inducing brown adipose tissue differentiation and thermogenesis in rodents [80], a role for H/O in non-shivering thermogenesis in birds has not been investigated.

We recently demonstrated that cFos expression in H/O neurons is affected by hypoxia in chick embryos and could be orchestrating the embryo response to hypoxia [81]. Similarly, H/O could be modulating chick embryo responses to hypothermia or other insults.

In summary, this study has demonstrated a very early production of H/O neuropeptides in chick embryo brains, and a relatively rapid early proliferation of H/O neurons in the hypothalamus. We suggest that the relatively early developmental production of H/O in chick embryos is related to the fact that they are self-sufficient and cannot depend on maternal production of H/O or other regulatory factors, or on nutrients of maternal origin.
